# Comprehensive Identification of Potential Crucial Genes and miRNA-mRNA Regulatory Networks in Papillary Thyroid Cancer

**DOI:** 10.1155/2021/6752141

**Published:** 2021-01-12

**Authors:** Ben-yu Nan, Guo-Feng Xiong, Zi-Rui Zhao, Xi Gu, Xin-Sheng Huang

**Affiliations:** ^1^Department of Otorhinolaryngology-Head and Neck Surgery, Zhongshan Hospital, Fudan University, Shanghai 200030, China; ^2^Department of Otorhinolaryngology-Head and Neck Surgery, Wenzhou Medical University, Affiliated Hospital 2, Wenzhou 325000, China; ^3^Department of Otolaryngology Head and Neck Surgery, Wenzhou Central Hospital, Wenzhou 325000, China; ^4^Department of Otolaryngology, Yueyang Hospital of Integrated Traditional Chinese and Western Medicine, Shanghai University of Traditional Chinese Medicine, Shanghai, 200437, China; ^5^Department of Otolaryngology-Head and Neck Surgery, The First Affiliated Hospital of Fujian Medical University, Fuzhou 350000, China

## Abstract

**Background:**

Thyroid cancer is the most common endocrine malignancy, with a recent global increase of 20% in age-related incidence. Ultrasonography and ultrasonography-guided fine-needle aspiration biopsy (FNAB) are the most widely used diagnostic tests for thyroid nodules; however, it is estimated that up to 25% of thyroid biopsies are cytologically inconclusive. Molecular markers can help guide patient-oriented and targeted treatment of thyroid nodules and thyroid cancer.

**Methods:**

Datasets related to papillary thyroid cancer (PTC) or thyroid carcinoma (GSE129562, GSE3678, GSE54958, GSE138042, and GSE124653) were downloaded from the GEO database and analysed using the Limma package of R software. For functional enrichment analysis, the Kyoto Encyclopedia of Genes and Genomes pathway analysis and Gene Ontology were applied to differentially expressed genes (DEGs) using the Metascape website. A protein-protein interaction (PPI) network was built from the STRING database. Gene expression, protein expression, immunohistochemistry, and potential functional gene survival were analysed using the GEPIA website, the Human Protein Atlas website, and the UALCAN website. Potential target miRNAs were predicted using the miRDB and Starbase datasets.

**Results:**

We found 219 upregulated and 310 downregulated DEGs, with a cut-off of *p* < 0.01 and ∣log FC | >1.5. The DEGs in papillary thyroid cancer were mainly enriched in extracellular structural organisation. At the intersection of the PPI network and Metascape MCODEs, the hub genes in common were identified as *FN1*, *APOE*, *CLU*, and *SDC2*. In the targeted regulation network of miRNA-mRNA, the hsa-miR-424-5p was found to synchronously modulate two hub genes. Survival analysis showed that patients with high expression of CLU and APOE had better prognosis.

**Conclusions:**

*CLU* and *APOE* are involved in the molecular mechanism of papillary thyroid cancer. The hsa-miR-424-5p might have the potential to reverse the processes of papillary thyroid cancer by modulating the hub genes. These are potential targets for the treatment of patients with papillary thyroid cancer.

## 1. Introduction

Thyroid cancer is the most common malignant tumour of the endocrine system. Its incidence has risen sharply worldwide in the past few years, as age-standardised incidence rates have increased by 20%, from 2.74 to 3.3 per 100,000 [1]. In the United States, in 2020 alone, the estimated numbers of new cases and deaths from thyroid cancer are 52,890 and 2,180, respectively [2]. Intriguingly, a parallel increase in global mortality is difficult to explain in the context of earlier diagnosis and better treatment [3].

According to criteria defined by the National Cancer Institute, there are several histological types of thyroid cancer: papillary, follicular, poorly differentiated, and anaplastic. Differentiated forms of papillary and follicular thyroid cancer contribute to more than 90% of all thyroid carcinomas [4]. It is estimated that 5 to 70% of adults could be diagnosed with thyroid nodules, by various clinical tests [5, 6], and the primary intention of their evaluation is to differentiate thyroid cancer from benign nodules. Most thyroid nodules can be determined by the commonly used diagnostic tests of ultrasonography and fine-needle aspiration biopsy (FNAB) [7]. However, it has been predicted that up to 25% of thyroid biopsies remain cytologically undefined, which can require diagnostic thyroid surgery [8].

Researchers have made much progress in discovering molecular mechanisms related to thyroid tumourigenesis, which can potentially be used as an adjunct in guiding clinical decisions. Somatic *BRAF* and *RAS* point mutations, as well as *RET/PTC* rearrangement, are the most recognised markers in this progression, involving the mitogen-activated protein kinase (MAPK) and PI3K/AKT signalling pathways [4]. This suggests the unprecedented possibility of more precise and effective approaches to the diagnosis and prognosis of thyroid cancer, based on the discovery of novel molecular markers.

This study used a high-throughput gene expression database to identify the potential molecular mechanisms of papillary thyroid cancer (PTC). In an attempt to elucidate the molecular mechanisms, lay a theoretical foundation, and provide well-defined therapeutic targets for the treatment of papillary thyroid cancer, we analysed differentially expressed genes (DEGs) and their regulatory relationships.

## 2. Materials and Methods

### 2.1. Microarray Datasets

**Figure 1:**
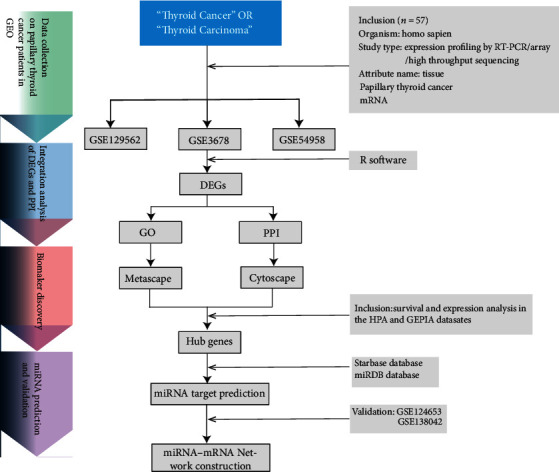
Study procedure flow chart for selecting of PTC genes.

The gene expression profiles of GSE129562, GSE3678, GSE54958, GSE138042, and GSE124653 were downloaded from the GEO database (https://www.ncbi.nlm.nih.gov/), and gene expression data for papillary thyroid cancer and normal thyroid tissue (partial paratumour normal thyroid tissue) were obtained. GSE129562, GSE3678, and GSE54958 were used for the identification of mRNA DEGs. GSE124653 and GSE138042 were used for the identification of differentially expressed miRNAs (DEMIs). The details and patient information of the datasets in the GEO are listed in Table 1. Our study procedure flowchart for searching papillary thyroid cancer target genes is shown in Figure 1.

### 2.2. Data Analysis and DEG Acquisition

After preprocessing and standardisation of raw biological data, the original datasets were analysed using the Limma package of R software. The DEGs were further analysed by taking the ∣log FC | >1.5 and *p* < 0.01 as thresholds. Volcano maps were drawn using R software. The intersection of the upregulated and downregulated genes was mapped using the Venn package (http://bioinformatics.psb.ugent.be/webtools/Venn/).

### 2.3. Functional Enrichment Analysis of DEGs

Gene Ontology (GO) and the Kyoto Encyclopedia of Genes and Genomes (KEGG) pathway analyses were enriched by Metascape [9], which provides a set of reliable, effective, and efficient tools to analyse and interpret bioinformatics studies. GO consists of three domains: molecular function (MF), cellular component (CC), and biological process (BP). DEGs were uploaded to Metascape for enrichment analysis. Terms with a *p* value < 0.01, a minimum count of 3, and an enrichment factor > 1.5 were collected and grouped into clusters based on their membership similarities.

### 2.4. Protein-Protein Interaction (PPI) Network of DEGs

The online database STRING 11.0 (http://string-db.org), a biological database and predictor of protein-protein interaction, was used to explore the PPI analysis of DEGs. An interaction with a combined score > 0.4 was considered statistically significant. Cytoscape (version 3.7.2) is a common source bioinformatics platform for the visualisation and analysis of molecular interaction networks. We also ran a PPI enrichment analysis using Metascape, with default parameters.

### 2.5. Hub Gene Confirmation and Analysis

The interaction coefficient between DEGs and hub genes was calculated using the plugin cytoHubba (version 0.1), and the hub genes were screened according to their degree.

Hub genes with ≥10 degrees were identified as high connectivity hub genes in the PPI network. In addition, the Metascape Molecular Complex Detection (MCODE) component was used to cluster a given network, based on topology, to find densely connected regions and identify the most densely connected networks. The cut-off criteria were the default values: degree cut-off = 2, node score cut-off = 0.2, Max depth = 100, and *K*‐score = 2.

### 2.6. Association of Gene Expression with the Survival of Thyroid Cancer Patients

The GEPIA website (http://gepia.cancer-pku.cn/) can provide quick customisation, based on TCGA data. The expression of target genes was analysed through the GEPIA website, and the prognosis of target genes was validated using the Human Protein Atlas website (https://www. http://proteinatlas.org/), which contains immunohistochemistry (IHC) data, location, staining intensity, quantity, and patient information regarding the type of cancer. The results from the two websites were used to identify each target gene. *p* < 0.05 was considered statistically significant.

### 2.7. Hub Gene-Related miRNA Prediction

MicroRNAs (miRNAs) are small, endogenous RNA molecules consisting of 21–25 nucleotides, and their highly conserved regions can target gene expression by binding to their 3′-untranslated regions (3′-UTRs). They play an important regulatory role in the aetiology of many animal and plant diseases and in pathophysiological and physiological functions. Each miRNA is supposed to be able to regulate multiple genes, via combinatorial and competitive interactions when bound to mRNA. To determine the potential interaction of miRNA-mRNA within the hub gene network, the online resources miRDB (http://mirdb.org/) and Starbase (http://starbase.sysu.edu.cn/) were employed for miRNA target prediction, and Cytoscape 3.7.1 was used to construct the miRNA–mRNA regulatory network.

## 3. Results

### 3.1. Identification of DEGs in Thyroid Cancer

**Figure 2:**
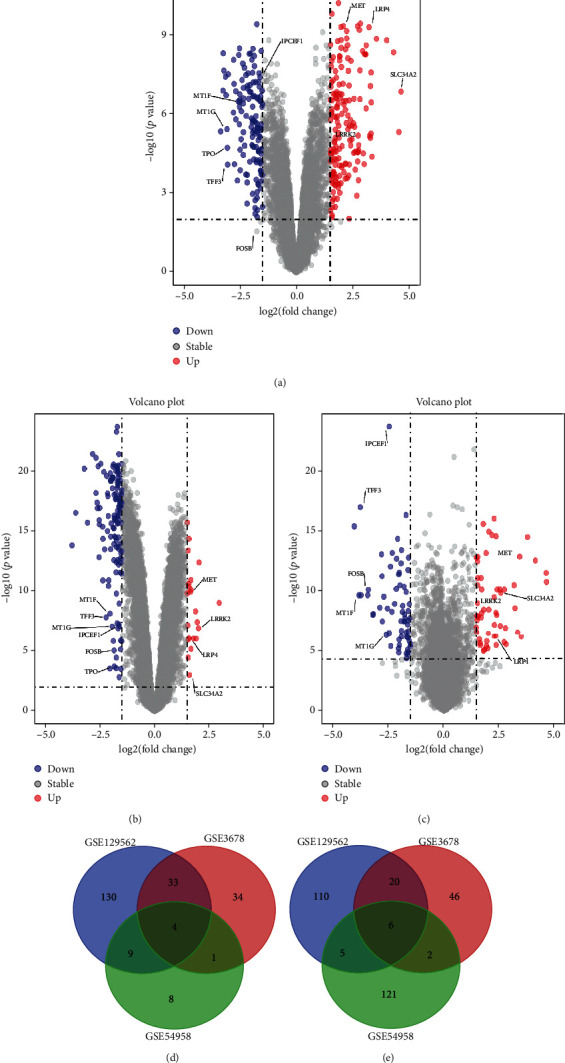
Identification of thyroid cancer mRNA and miRNA DEGs. (a–c) Volcano map of GSE129562, GSE3678, and GSE54958, respectively. (d, e) Four upregulated and six downregulated DEGs were selected based on the intersection between upregulated and downregulated genes in GSE129562, GSE3678, and GSE54958.

The gene expression profiles of GSE129562, GSE3678, GSE54958, GSE138042, and GSE124653 were downloaded from the GEO database for papillary thyroid carcinoma and paired, normal thyroid tissue. As shown in Figure 2, 13530, 20188, and 18837 DEGs from GSE129562, GSE3678, and GSE54958 were extracted, respectively (Figures 2(a)–2(c)).

R cluster analysis software (∣log FC | >1.5 and *p* value < 0.01 as the cut-off) found that, in diseased tissue compared with the paired normal thyroid tissue, there were 219 upregulated genes and 310 downregulated genes. The common DEGs in the three datasets were identified using Venn diagram software (Figures 2(d) and 2(e)). The results showed a total of 10 common DEGs, among which 6 were downregulated and 4 were upregulated.

### 3.2. DEGs, GO, and KEGG Pathway Analysis in Thyroid Cancer

**Figure 3:**
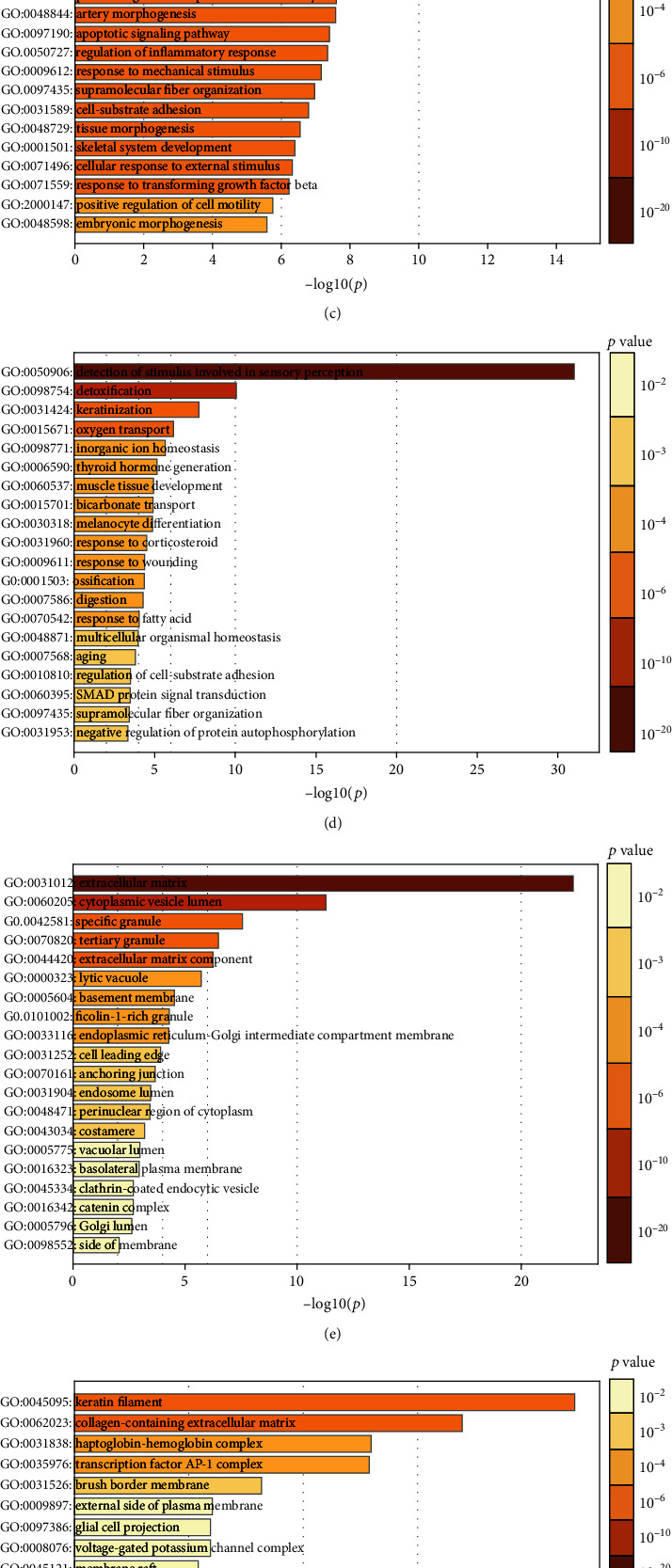
GO Function enrichment analysis and KEGG pathway analysis of DEGs in thyroid cancer by Metascape. (a, b) MF analysis of upregulated and downregulated DEGs, respectively. (c, d) BP analysis of upregulated and downregulated DEGs, respectively. (e, f) CC analysis of upregulated and downregulated DEGs, respectively. (g, h) KEGG of upregulated and downregulated DEGs, respectively.

In an attempt to analyse the biological classification of DEGs, the Metascape website was used for functional and pathway enrichment analysis. BP enrichment showed that the increase in DEGs was mainly concentrated in extracellular structure organisation, myeloid leukocyte activation, blood vessel development, response to wounding, and regulation of cell adhesion (Figure 3(a)), while the decrease in DEGs was mainly concentrated in the detection of stimuli involved in sensory perception, detoxification, keratinisation, and oxygen transport (Figure 3(b)). GO analysis showed that the MF changes of upregulated DEGs were mainly concentrated in structural constituents of the extracellular matrix, glycosaminoglycan binding, proteoglycan binding, protease binding, and cell adhesion molecular binding (Figure 3(c)), while downregulated DEGs in MF were mainly concentrated in olfactory receptor activity and oxygen carrier activity (Figure 3(d)). Changes in the CC of upregulated DEGs were mainly enriched in the extracellular matrix, cytoplasmic vesicle lumen, specific granule, tertiary granule, and extracellular matrix components (Figure 3(e)), whereas downregulated DEGs in CC were mainly found in the following GO terms: keratin filament and collagen-containing extracellular matrix (Figure 3(f)).

KEGG pathway analysis showed that the DEGs were primarily concentrated in ECM-receptor interaction, complement and coagulation cascades, focal adhesion, cell adhesion molecules, (CAMs), transcriptional misregulation in cancer, pathways in cancer, the p53 signalling pathway, the TGF-beta signalling pathway, thyroid hormone synthesis, and the NF-kappaB signalling pathway (Figures 3(g) and 3(h)).

### 3.3. Identification of Hub Genes

**Figure 4:**
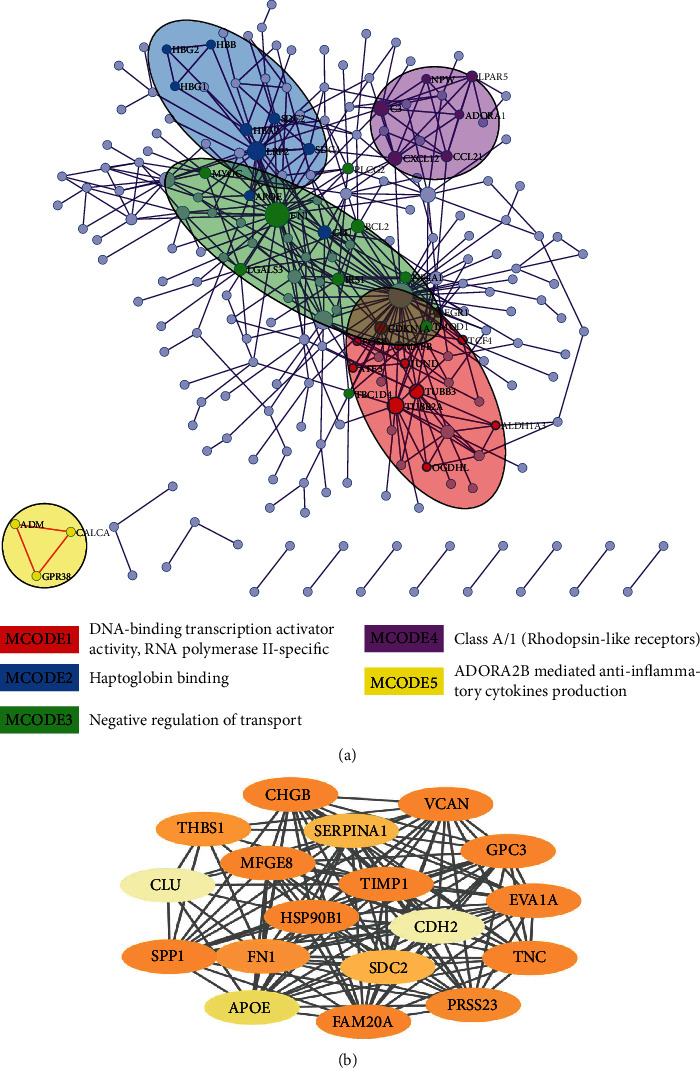
MCODE enrichment analysis by Metascape. (a) PPI interaction network. The MCODE algorithm was applied to clustered enrichment ontology terms to identify neighborhoods where proteins are densely connected. Each MCODE network is assigned a unique color. GO enrichment analysis was applied to each MCODE network. See Supplementary Table S1 for more details. Red, blue, green, violet, and orange colors indicate modules 1, 2, 3, 4, and 5, respectively. (b) Additionally, 18 most significant genes of four models of MCC, degree, DMNC, and MNC were identified using the cytoHubba plugin in the Cytoscape.

To clarify the significant correlation of DEGs in papillary thyroid carcinoma, we combined the 529 DEGs using the STRING online database (http://string-db.org) and Cytoscape software. The PPI network of DEGs was constructed, and the most significant module was obtained from Cytoscape. The top 30 genes were screened by selecting the models of maximal clique centrality (MCC), degree, density of maximum neighbourhood component (DMNC), and maximum neighbourhood component (MNC) in the cytoHubba plugin (Table 2). The top 30 genes in the four models contain 18 common genes: *PRSS23*, *TNC*, *HSP90B1*, *MFGE8*, *GPC3*, *CHGB*, *VCAN*, *FN1*, *FAM20A*, *THBS1*, *SPP1*, *APOE*, *SERPINA1*, *CLU*, *TIMP1*, *SDC2*, *EVA1A*, and *CDH2* (Figure 4(b)).

Additionally, Metascape online was applied to discover the hub clusters in the network with the MCODE component based on PPI enrichment analysis. In total, 15 modular MCODEs were extracted from the 529 DEGs, which included 38 hub genes and four seed genes: *ADORA*, *HBB*, *EGR1*, and *GPR83* (Figure 4 and Table S1). We intersected the 18 hub genes extracted by cytoHubba with 38 hub genes identified by Metascape and found four common hub genes: *FN1*, *APOE*, *CLU*, and *SDC2*.

### 3.4. Analysis of Hub Genes by Survival Analysis and Expression

**Figure 5:**
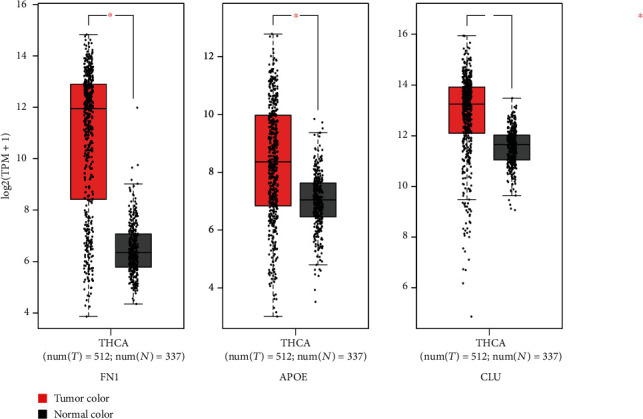
Expression analysis of FN1, APOE, and CLU in THCA based on GEPIA. |log2 FC| cut-off: 1; *p* value cut-off: 0.01.

**Figure 6:**
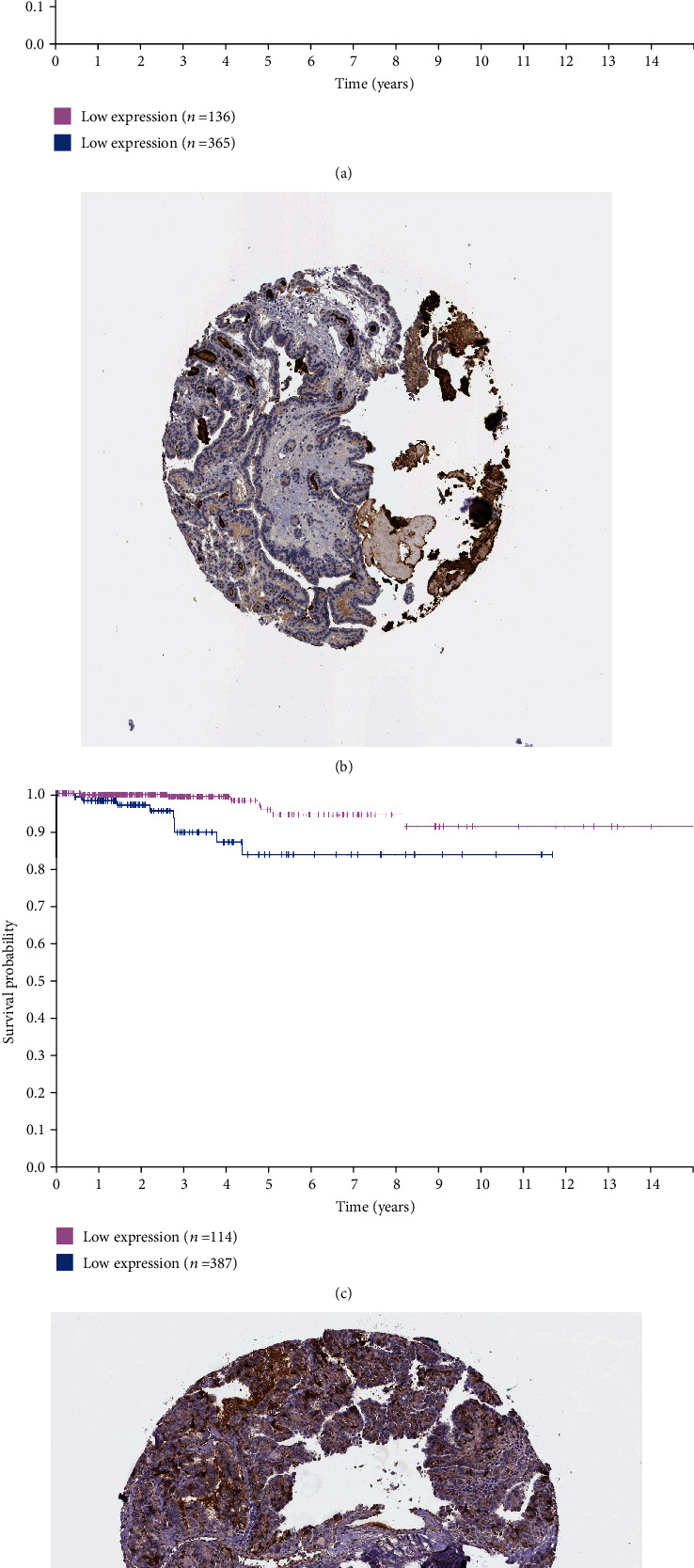
Survival analysis and protein expression of the 2 target genes in THCA based on the Human Protein Atlas. (a, b) CLU. (c, d) APOE. *p* < 0.05 was considered statistically significant.

A survival analysis of the hub genes was performed using the Human Protein Atlas online tool, and expression was analysed using the GEPIA website, which helped us to investigate the correlation between hub genes and survival of patients with thyroid cancer. We found that, in thyroid cancer, the expressions of FN1, APOE, and CLU were statistically significant (Figure 5). Although there was no significant correlation between the high expression of FN1 and the survival status of patients with thyroid cancer (*p* > 0.05), patients with high expression of CLU (Figure 6(a)) and APOE (Figure 6(c)) tended to live longer. Thus, we chose APOE and CLU as our target genes for better prognosis.

### 3.5. The Biological Roles of the Target Genes in Tumours

**Figure 7:**
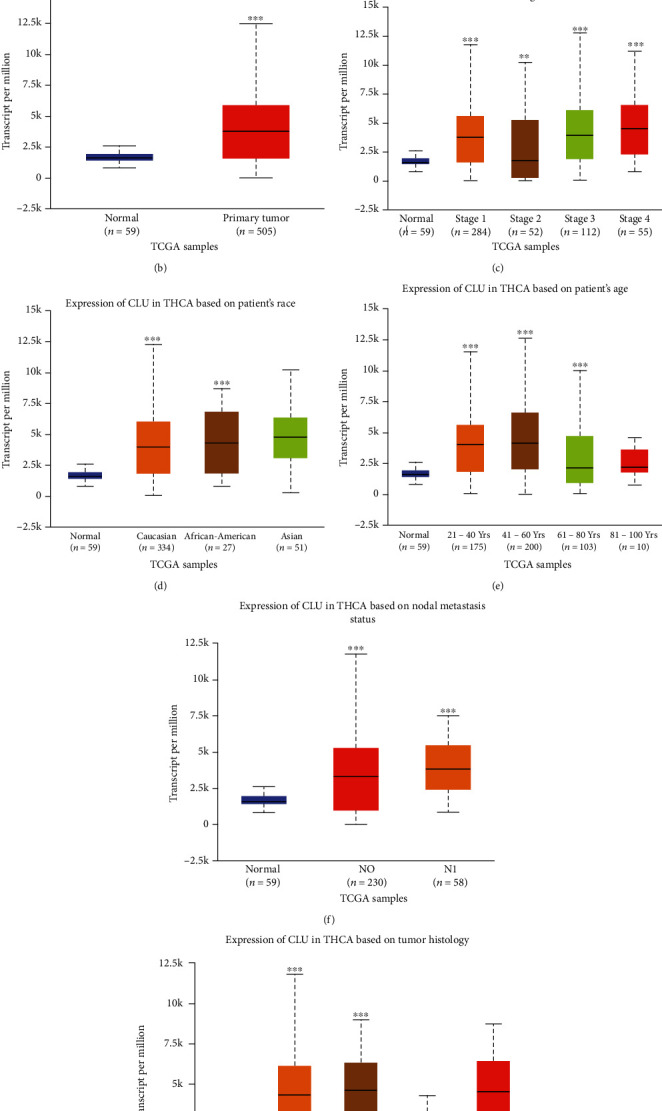
The biological role of CLU in tumours. (a) Expression of CLU in various tumours on the GEPIA website. (b) Expression of CLU in THCA based on sample type. (c) Expression of CLU in THCA based on individual cancer stages. (d, e) Expression of CLU in THCA based on a patient's race and age. (f) Expression of CLU based on nodal metastasis status of THCA. (g) Expression of CLU based on tumour histology of THCA. ^∗^*p* < 0.05, ^∗∗^*p* < 0.01, and ^∗∗∗^*p* < 0.001.

**Figure 8:**
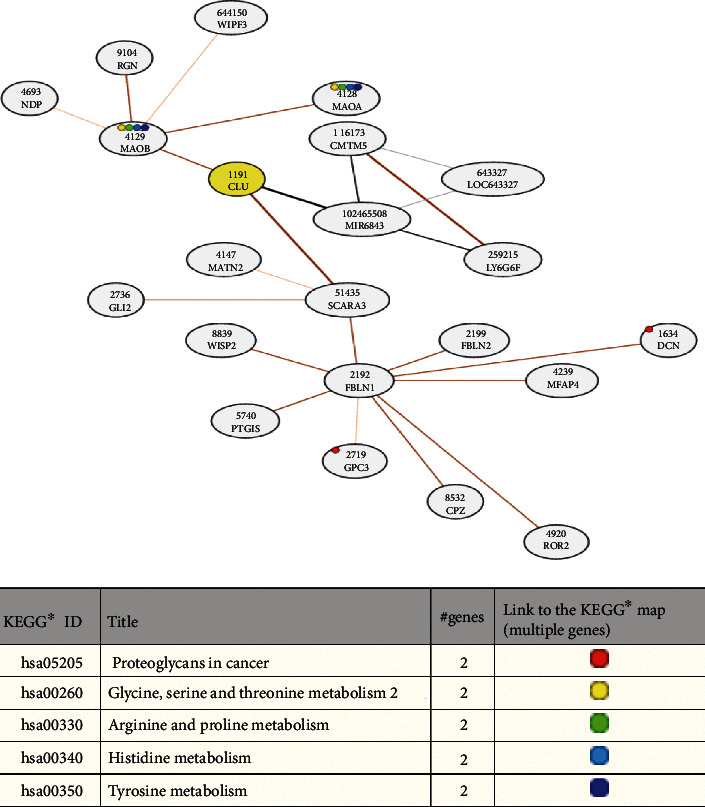
Network for CLU coexpressed genes and their KEGG enrichment analysis.

To study whether the target genes play a role in the oncogenesis of other tumours, we analysed their differential expression in normal tissues and in various carcinomas, using the GEPIA website. The analysis showed that *CLU* was upregulated in a variety of tumours, including lymphoid neoplasm diffuse large B-cell lymphoma (DLBC), glioblastoma multiforme (GBM), kidney renal papillary cell carcinoma (KIRP), acute myeloid leukaemia (AML), brain lower grade glioma (LGG), ovarian serous cystadenocarcinoma (OV), thyroid carcinoma (THCA), and thymoma (THYM) (Figure 7(a)). This implies that, for some tumours, the underlying mechanisms of CLU in regulating their occurrence and development are identical. We confirmed that CLU was highly expressed in THCA, using the UALCAN website (http://ualcan.path.uab.edu/) (Figure 7(b)). Differential expression of CLU was found to vary according to the cancer stage (Figure 7(c)), the patient's race (Figure 7(d)), the patient's age (Figure 7(e)), nodal metastatic status (Figure 7(f)), and histological subtype (Figure 7(g)). To investigate the potential molecular mechanisms of CLU, coexpressed genes were identified via the COXPRESdb website (http://coxpresdb.jp). This analysis showed that CLU-related genes were primarily concentrated in the proteoglycans in cancer and in glycine, serine and threonine metabolism, arginine and proline metabolism, histidine metabolism, and tyrosine metabolism (Figure 8). We discovered that CLU had a strong coexpressive relationship with MIR6843, SCARA3, and MAOB (Figure 8).

**Figure 9:**
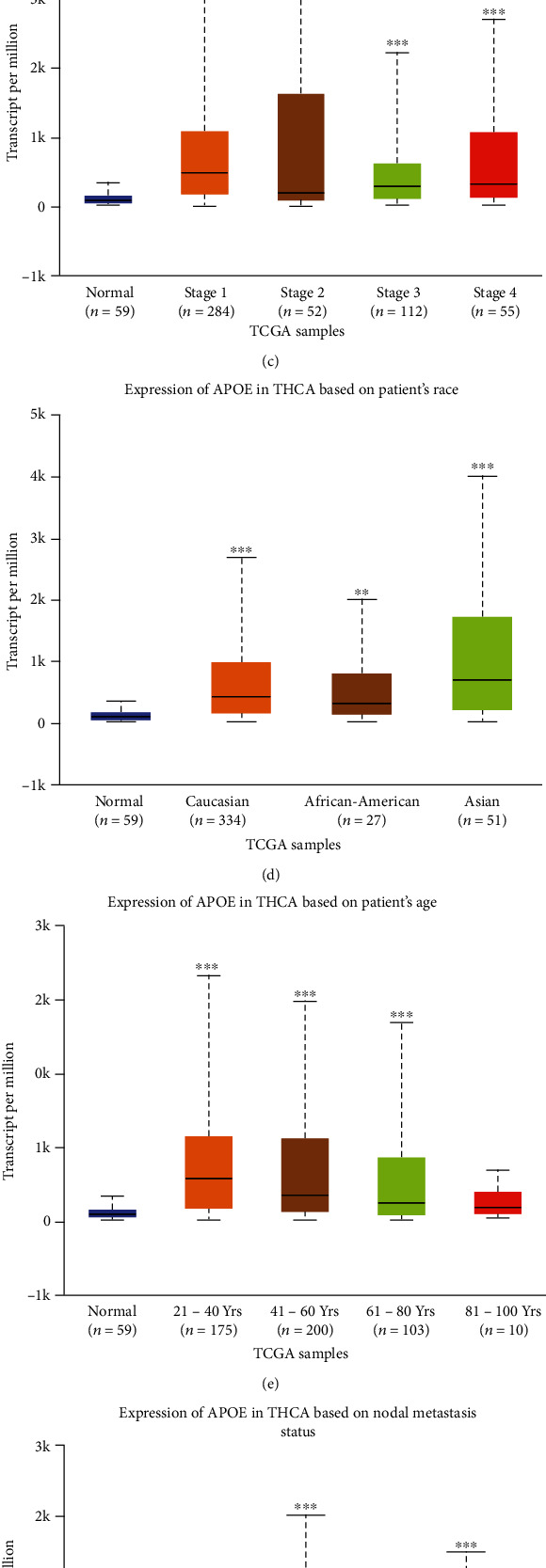
The biological role of APOE in tumours. (a) Expression of CLU in various tumours on the GEPIA website. (b) Expression of CLU in THCA based on sample type. (c) Expression of CLU in THCA based on individual cancer stages. (d, e) Expression of CLU in THCA based on a patient's race and age. (f) Expression of CLU based on nodal metastasis status of THCA. (g) Expression of CLU based on tumour histology of THCA. ^∗^*p* < 0.05, ^∗∗^*p* < 0.01, and ^∗∗∗^*p* < 0.001.

**Figure 10:**
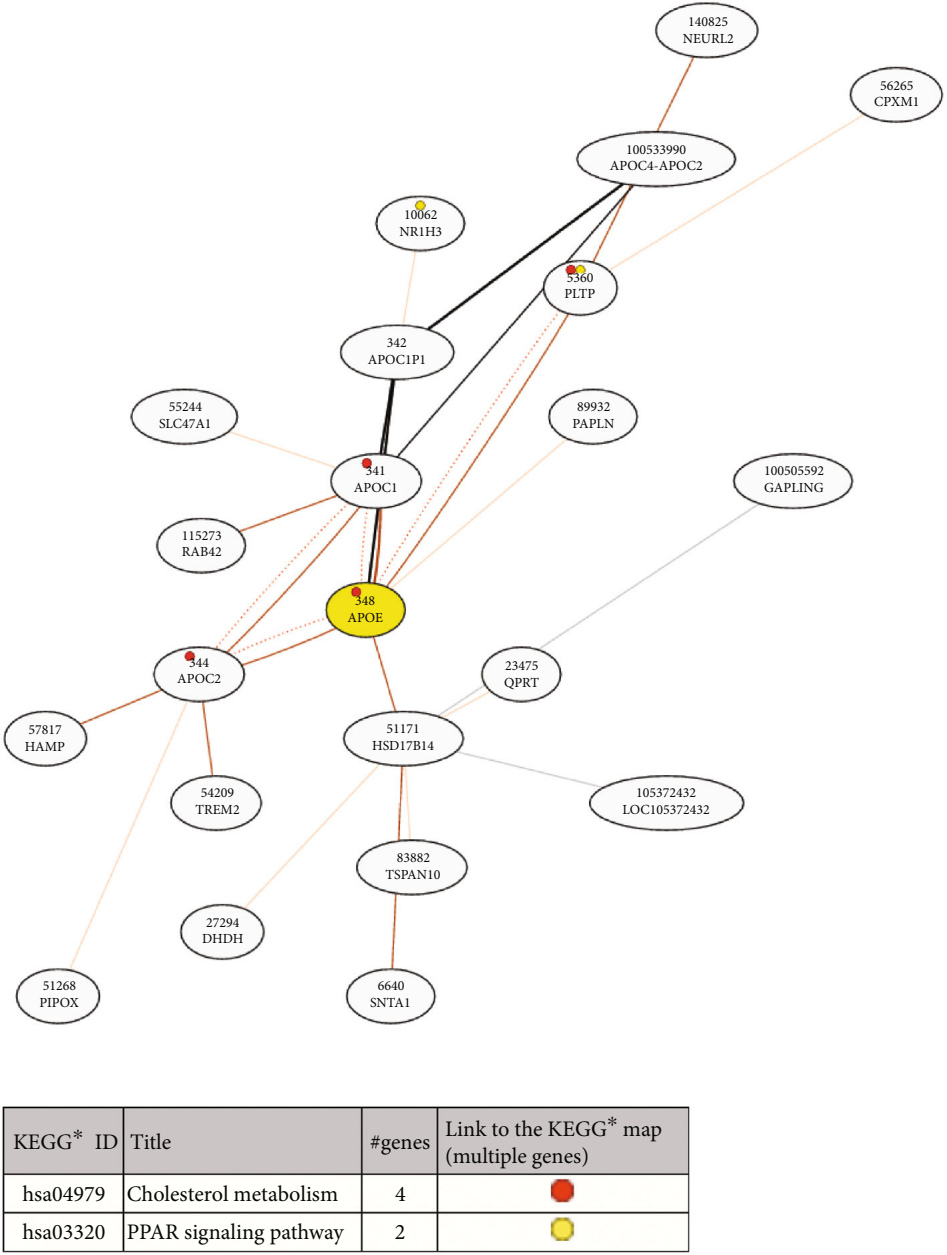
Network for APOE coexpressed genes and their KEGG enrichment analysis.

To investigate whether the *APOE* gene plays an oncogenic role in other tumours, we analysed its differential expression in normal tissues and in various tumours, through the GEPIA website. This showed that APOE was upregulated in a variety of tumours, including DLBC, oesophageal carcinoma (ESCA), GBM, head and neck squamous cell carcinoma (HNSC), LGG, liver hepatocellular carcinoma (LIHC), pancreatic adenocarcinoma (PAAD), prostate adenocarcinoma (PRAD), skin cutaneous melanoma (SKCM), stomach adenocarcinoma (STAD), testicular germ cell tumours (TGCTs), THCA, THYM, uterine corpus endometrial carcinoma (UCEC), and uterine carcinosarcoma (UC) (Figure 9(a)). This implies that there is a common underlying mechanism for APOE in regulating tumour occurrence and development. We confirmed that APOE was highly expressed in THCA, via the UALCAN website (Figure 9(b)). Differential expression of APOE was found in patients with different cancer stages (Figure 9(c)) and of different race (Figure 9(d)), age (Figure 9(e)), nodal metastatic status (Figure 9(f)), and histological subtype (Figure 9(g)). To investigate the potential molecular mechanisms of APOE, the genes coexpressed with APOE were identified via the COXPRESdb website (http://coxpresdb.jp), and analysis showed that APOE-related genes are primarily concentrated in cholesterol metabolism and the PPAR signalling pathway (Figure 10). We discovered that APOE has a strong coexpressive relationship with APOC1, APOC1P1, APOC2, HSD17B14, PLTP, and PAPLN (Figure 10).

### 3.6. miRNA-mRNA Network Construction

**Figure 11:**
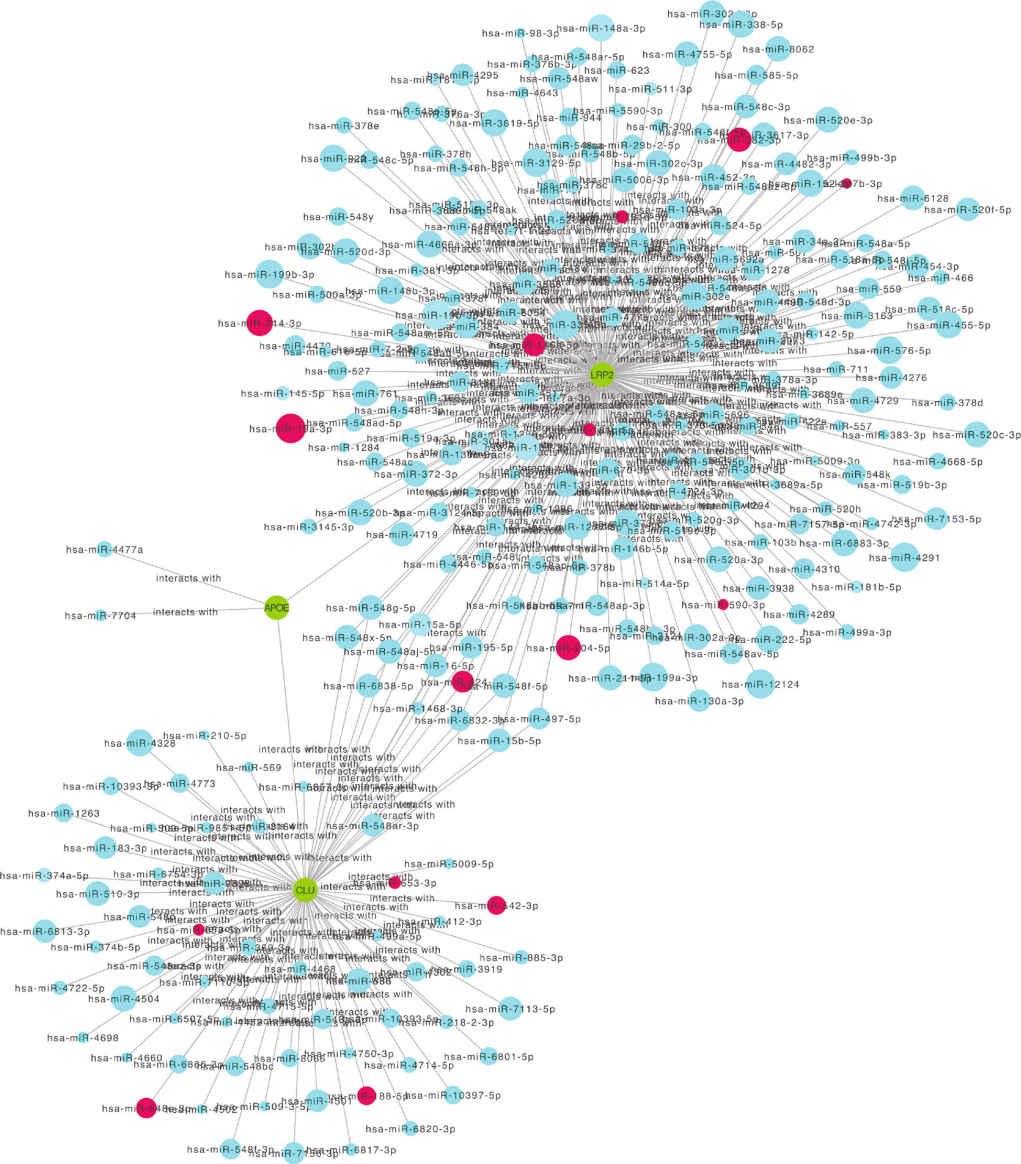
The construction of miRNA-mRNA network of hub genes in THCA. The red circles predicted the potential miRNAs that can be validated in GSE124653 and GSE138042. The green circular nodes represent mRNAs. The size of the nodes is equal to the target score of interaction between miRNAs and mRNA.

The miRDB and Starbase databases were used for miRNA target prediction and functional annotations, which are involved in regulating the transcription of target genes. The probability scores were predicted using the miRDB database, and a high score of miRNA-mRNA reflected a close potential function of miRNA in regulating the target messenger. We filtered the predicted miRNAs that were identified by at least one of eight miRNA target prediction programs including PITA, miRmap, miRanda, microT, picTar, RNA22, PITA, and targetScan in the Starbase. The overlapped predicted miRNAs between the miRDB database and Starbase database were chosen to construct a miRNA-mRNA network using Cytoscape software with a cut-off > 50 (Figure 11).

**Figure 12:**
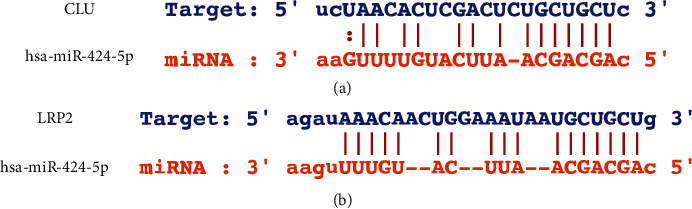
The potential binding sites for hsa-miR-424-5p was identified at the 3′-UTR of CLU (a) and LRP2 (b).

We constructed a predicted miRNA network for CLU, LRP2, and APOE, since APOE and CLU were connected via LRP2 in the MCODE of the Metascape website. Interestingly, 16 miRNAs were validated using GSE124653 and GSE138042: hsa-miR-542-3p, hsa-miR-424-5p, hsa-miR-653-5p, hsa-miR-146b-5p, hsa-miR-181d-5p, hsa-let-7b-3p, hsa-miR-19a-3p, hsa-miR-653-3p, hsa-miR-582-3p, hsa-miR-188-5p, hsa-miR-204-5p, hsa-miR-181c-5p, hsa-miR-548e-3p, hsa-miR-15a-5p, hsa-miR-214-3p, and hsa-miR-590-3p. Interestingly, hsa-miR-424-5p can regulate both CLU and LRP2 simultaneously and may play a crucial role in the progression of carcinogenesis. The potential binding sites for hsa-miR-424-5p were validated at the 3′-UTR region of CLU and LRP2 using the miRanda program in the Starbase (Figure 12).

## 4. Discussion

Thyroid nodules are extremely common and are often found in asymptomatic patients who are being evaluated for other conditions [6, 10]. Thyroid nodules are commonly evaluated by tests involving thyroid function, ultrasound examination, and FNAB of selected nodules. However, approximately 25% of FNA cytology samples yield more than two types of indeterminate cytological diagnosis [11]. Moreover, the insufficient selection of thyroid nodules for biopsy can also lead to a missed diagnosis of thyroid cancer. The management of patients with indeterminate nodules is challenging, since the estimated risk of thyroid cancer is unpredictable (5–75%) [12–14].

Molecular cytology diagnosis has been applied to multiplatform detection of DNA, mRNA, and miRNA, which can help to identify inconclusive thyroid nodules and further improve the preoperative risk management of benign nodules with undetermined cytology [15]. Researchers have reviewed the top 12 recommended markers, which include those well-studied (*MET*, *TFF3*, *SERPINA1*, *TIMP1*, *FN1*, and *TPO*) as well as those that are relatively novel (*TGFA*, *QPCT*, *CRABP1*, and *PROS1*) [16].

To identify DEGs and target genes, we analysed the differences in gene profile between papillary thyroid cancer tissue and normal thyroid tissue. Molecular mechanisms and regulatory relationships with papillary thyroid cancer were identified, based on the high-throughput analysis of the gene expression database. Moreover, a theoretical foundation for the diagnosis of papillary thyroid cancer and accurate personal therapeutic targets are provided by our analysis. DEGs may be useful for GO analysis, and target genes can facilitate clinical studies, following consideration of their clinical relevance. In this study, 529 integrated DEGs were found in papillary thyroid cancer, using a comprehensive analysis of GEO (GSE129562, GSE3678, and GSE54958). GO (BP, CC, and MF) analysis was then performed on the 529 integrated DEGs. The DEG enrichment analysis yielded many terms concentrated in BP, CC, and MF. These results indicate that these DEGs are involved in the extracellular matrix of thyroid cancer cells. The KEGG pathway analysis showed that DEGs were primarily concentrated in ECM-receptor interaction, complement and coagulation cascades, cell adhesion molecules (CAMs), transcriptional misregulation in cancer, pathways in cancer, thyroid hormone synthesis, mineral absorption, and tyrosine metabolism. The ECM is highly dynamic since it is constantly deposited, reshaped, and degraded throughout development until maturity, to maintain tissue homoeostasis [17]. The composition and organisation of the ECM are spatiotemporally regulated to control cell behaviour and differentiation, and the dysregulation of ECM dynamics can lead to the development of diseases such as cancer [18]. Therefore, investigating this pathway will lead to a better understanding of the proliferation and invasion of papillary thyroid cancer and will help to predict tumour progression.

We built a PPI network with 529 integrated DEGs, and 18 hub genes were identified. Additionally, the MCODE component of Metascape online was also applied, and 38 hub genes were discovered. We then intersected the 18 hub genes and 38 hub genes identified by different methods; four hub genes (*FN1*, *APOE*, *CLU*, and *SDC2*) were found in common. They affect tumourigenesis and progression, mainly by affecting cell adhesion, migration, and apoptosis. These hub genes could be used as therapeutic targets in the management of papillary thyroid cancer and inconclusive thyroid nodules. Then, these 4 hub genes were subjected to a prognostic analysis using the GEPIA and Human Protein Atlas websites. Surprisingly, CLU and APOE expression showed strong relationships with the prognosis of patients with papillary thyroid cancer. Therefore, we chose *CLU* and *APOE* as target genes for further analysis.

The protein encoded by *CLU* is a secreted chaperone that may be involved in several basic biological events, such as cancer initiation and progression, and neurodegenerative disorders [19, 20]. This involvement suggests that CLU might play an important role in cell death, cell cycle regulation, DNA repair, cell adhesion, tissue re-modelling, lipid transportation, membrane recycling, and immune system regulation [19, 20]. Indeed, CLU is selectively overexpressed and strongly associated with increased tumourigenicity, metastatic potential, and resistance to chemotherapy [21–24]. It has been found that CLU mRNA and protein are overexpressed in several human cancers, including cancer of the prostate, breast, lung, kidney, ovary, colon, and endometrial tissues [25–33]. Overexpressions of CLU in DLBC, GBM, KIRP, LAML, LGG, OV, THCA, and THYM patients were found by using the GEPIA website, which was consistent with our main analysis. It has been reported that apoptotic triggers can upregulate CLU, which acts as a cell survival gene, and can affect cell resistance to apoptosis in carcinomas [34]. CLU was found within distinct, bipartite patches on the basolateral plasma membranes of cultured porcine thyrocytes, suggesting that it is a component of cell-adhesion complexes and is involved in cell-cell and cell-matrix interactions [35]. Our analysis showed that CLU is differentially expressed in patients according to cancer stage, race, age, nodal metastasis, and histological subtype. We also found that CLU has a strong coexpressive relationship with MIR6843, SCARA3, and MAOB.

APOE is an apolipoprotein associated with lipid particles, mainly mediating lipid transport between organs via the plasma and interstitial fluids [36–38]. In addition, APOE is involved in innate and adaptive immune responses, such as controlling the survival of myeloid-derived suppressor cells [39]. It has also been reported that APOE is related to a variety of biological cellular events, such as cell proliferation, migration, adhesion, and immunoregulation, in a tissue-dependent manner [40]. Many studies have identified a link between *APOE* and carcinogenesis via metabolic mechanisms, including DNA synthesis, *β*-catenin localisation, cell proliferation, antioxidant function, angiogenesis, and metastasis [41, 42]. Many reports have shown that APOE can be overexpressed in human malignancies, including cancers of the bladder, breast, and ovary, colorectal cancer, renal cell carcinoma, and gastric cancer [42–47]. It is consistent with these findings that the levels of APOE in DLBC, ESCA, GBM, HNSC, LGG, LIHC, PAAD, PRAD, SKCM, STAD, TGCT, THCA, THYM, UCEC, and UC patients were found to be overexpressed, using the GEPIA website. Although *APOE* mRNA is expressed in significant quantities in various normal human organs [40, 48], it is almost undetectable in the thyroid and the expression of APOE protein is negative [49]. However, it has also been reported that APOE was highly expressed in THCA, via the UALCAN website. This suggests that high APOE expression may be an important characteristic of thyroid cancer. Our analysis showed that APOE is differentially expressed in THCA patients according to cancer stage, race, age, nodal metastasis, and histological subtype. We also found that APOE has a strong coexpressive relationship with APOC1, APOC1P1, APOC2, HSD17B14, PLTP, and PAPLN.

It was found that both *APOE* and *CLU* could delay the initiation time of amyloid growth kinetics in a concentration-dependent manner, acting as extracellular chaperones to inhibit amyloid-*β* deposition in patients with sporadic cerebral amyloid angiopathy [50]. Additionally, studies have shown that concentrations of APOA1, APOE, and CLU were differentially expressed in cervical squamous cell carcinoma patients when compared to those with benign lesions and were associated with the histological classification or the processing of the cervical lesion [51]. Interestingly, we found that *APOE* correlated with *CLU* via *LRP2* in MCODE2, and *CLU* and *LRP2* had common predicted miRNAs, which implicated hub genes and their potential miRNAs that might affect thyroid cancer proliferation and invasion through the extracellular matrix.

## 5. Conclusion

We found that *APOE* and *CLU* were the hub genes regulating proliferation and invasion through the extracellular matrix in papillary thyroid cancer. We speculated that the predicted hsa-miR-424-5p might have the potential to reverse the processes of papillary thyroid cancer by modulating the hub genes. These are potential targets for the treatment of patients with papillary thyroid cancer.

## Figures and Tables

**Table 1:** List of details and patient information of the datasets in GEO.

GEO ID	GSE129562	GSE54958	GSE3678	GSE124653	GSE138042
Total no. of patients	8	Not mentioned	Not mentioned	Not mentioned	Not mentioned
Total no. of samples	16	38	7	29	81
No. of nontumour samples	8	7	7	3	57
Tumour types	Papillary thyroid cancer	Papillary thyroid cancer	Papillary thyroid cancer	Thyroid papillary/follicular carcinoma	Thyroid cancer
Grading of tumours (TNM stage)	Stage I-II	Not mentioned	Not mentioned	Primary tumor	Primary tumor
Pathological grade	Not mentioned	Not mentioned	Not mentioned	No subdivision	No subdivision

**Table 2:** List of top 30 genes of 4 modes of MCC, DMNC, MNC, and degree in cytoHubba plugin.

MCC	DMNC	MNC	Degree
FN1	SPP1	FN1	FN1
SERPINA1	CDH2	SERPINA1	C3
TIMP1	EVA1A	C3	SERPINA1
SDC2	CHGB	TIMP1	TIMP1
GPC3	PRSS23	APOE	SDC2
VCAN	MFGE8	SDC2	APOE
APOE	FAM20A	GPC3	GPC3
TNC	HSP90B1	VCAN	SPP1
C3	VCAN	TNC	CDH2
SPP1	TNC	HSP90B1	VCAN
CDH2	GPC3	SPP1	TNC
HSP90B1	SDC2	CDH2	HSP90B1
EVA1A	MMRN1	EVA1A	EVA1A
CHGB	TMSB4X	CHGB	CHGB
PRSS23	PROS1	PRSS23	PRSS23
MFGE8	TIMP1	MFGE8	MFGE8
FAM20A	TMC6	FAM20A	FAM20A
CFD	ATP11A	CFD	THBS1
CLU	ALDH3B1	CLU	SDC4
THBS1	OLR1	THBS1	LYZ
MMRN1	APOE	CD47	CD36
TMSB4X	CLU	SDC4	DCN
PROS1	COL8A1	LYZ	CFD
CD36	P4HA2	QPCT	CD47
CD47	COL5A2	MMRN1	PLAUR
PLAUR	THBS1	TMSB4X	JUN
PLAU	SERPINA1	PROS1	CLU
TMC6	FN1	CD36	QPCT
ATP11A	COL23A1	PLAUR	COL3A1
ALDH3B1	DCN	PLAU	ITGA2

## Data Availability

GSE129562, GSE3678, GSE54958, GSE138042, and GSE124653 were downloaded from the Gene Expression Omnibus.

## Supplementary Materials

Supplementary Materials Table S1: gene list of 5 MCODE modules shown in Figure 3(b).
