# Supplementary material for: Comprehensive Identification of Potential Crucial Genes and miRNA-mRNA Regulatory Networks in Papillary Thyroid Cancer
Source: Biomed Res Int. 2021 Jan 12;2021:6752141. doi: 10.1155/2021/6752141 (PMC7817291; doi:10.1155/2021/6752141)
Supplement: Supplementary Materials — Table S1: gene list of 5 MCODE modules shown in Figure 3(b). [file 6752141.f1.docx]

Table S1| Gene list of 5 MCODE modules shown in Figure 3B.

| Cluster | Gene | Score | Type |
| --- | --- | --- | --- |
| 1 | \| CDKN1A \| \| --- \| | 1.18181818 | Clustered |
| 1 | TUBB3 | 1.18181818 | Clustered |
| 1 | TCF4 | 1.18181818 | Clustered |
| 1 | EGR1 | 1.18181818 | Seed |
| 1 | ALDH1A3 | 1.18181818 | Clustered |
| 1 | OGDHL | 1.18181818 | Clustered |
| 1 | JUND | 1.18181818 | Clustered |
| 1 | TUBB2A | 1.18181818 | Clustered |
| 1 | ATF3 | 1.18181818 | Clustered |
| 1 | MAFB | 1.18181818 | Clustered |
| 1 | FOSB | 1.18181818 | Clustered |
| 2 | \| HBA2 \| \| --- \| | 1.55555556 | Clustered |
| 2 | HBB | 1.55555556 | Seed |
| 2 | HBG1 | 1.55555556 | Clustered |
| 2 | HBG2 | 1.55555556 | Clustered |
| 2 | CLU | 1.55555556 | Clustered |
| 2 | LRP2 | 1.55555556 | Clustered |
| 2 | SDC4 | 1.55555556 | Clustered |
| 2 | SDC2 | 1.55555556 | Clustered |
| 2 | APOE | 1.55555556 | Clustered |
| 3 | \| FN1 \| \| --- \| | 1.22222222 | Clustered |
| 3 | MYOC | 1.22222222 | Clustered |
| 3 | LGALS3 | 1.22222222 | Clustered |
| 3 | PLCG2 | 1.22222222 | Clustered |
| 3 | BCL2 | 1.22222222 | Clustered |
| 3 | IRS1 | 1.22222222 | Clustered |
| 3 | TBC1D4 | 1.22222222 | Clustered |
| 3 | NR4A1 | 1.22222222 | Clustered |
| 3 | TMOD1 | 1.22222222 | Clustered |
| 4 | \| CCL21 \| \| --- \| | 2.5 | Clustered |
| 4 | C3 | 2.5 | Clustered |
| 4 | CXCL12 | 2.5 | Clustered |
| 4 | NPW | 2.5 | Clustered |
| 4 | LPAR5 | 2.5 | Clustered |
| 4 | ADORA1 | 2.5 | Seed |
| 5 | \| ADM \| \| --- \| | 1 | Clustered |
| 5 | CALCA | 1 | Clustered |
| 5 | GPR83 | 1 | Seed |
